# CD38^hi^CD19^dim^ cells in lymph nodes predict favorable prognosis in patients with stage III melanoma receiving adjuvant PD-1-blockade

**DOI:** 10.3389/fonc.2026.1815008

**Published:** 2026-05-04

**Authors:** Ieva Ailte, Sudhir Kumar Chauhan, Lina Prasmickaite, Assia V. Bassarova, Amanda Poissonnier, Marike Feenstra, Marta Nyakas, Bjarne Johannessen, Truls Ryder, Robert Hermann, Lars Frich, Arild Holth, Else Marit Inderberg, Henrik Jespersen, Vivi Ann Flørenes, Jon Amund Kyte, Gunhild M. Mælandsmo, Eivind Valen Egeland

**Affiliations:** 1 Department of Tumor Biology, Institute for Cancer Research, The Norwegian Radium Hospital, Oslo University Hospital, Oslo, Norway; 2 Department of Pathology, Division of Laboratory Medicine, Norwegian Radium Hospital, Oslo University Hospital, Oslo, Norway; 3 Department of Cancer Immunology, Institute for Cancer Research, The Norwegian Radium Hospital, Oslo University Hospital, Oslo, Norway; 4 Department of Cell, Developmental & Cancer Biology, Oregon Health & Science University, Portland, OR, United States; 5 Department of Oncology, The Norwegian Radium Hospital, Oslo University Hospital, Oslo, Norway; 6 Department of Molecular Oncology, Institute for Cancer Research, The Norwegian Radium Hospital, Oslo University Hospital, Oslo, Norway; 7 Department of Oncologic Plastic Surgery, The Norwegian Radium Hospital, Oslo University Hospital, Oslo, Norway; 8 Section for Cellular Therapy, Department of Oncology, The Norwegian Radium Hospital, Oslo University Hospital, Oslo, Norway; 9 Institute of Clinical Medicine, University of Oslo, Oslo, Norway; 10 Department of Medical Biology, Faculty of Health Sciences, The Arctic University of Norway-University of Tromsø, Tromsø, Norway

**Keywords:** CD103^+^PD-1^+^CD8^+^ T cells, CD38^hi^CD19^dim^, immune checkpoint inhibitors, PD1-blockade, plasmablast-like cells, stage III melanoma

## Abstract

**Background:**

Adjuvant immune checkpoint inhibitors (ICI) have improved survival in stage III cutaneous melanoma, yet many patients do not benefit. The tumor microenvironment is pivotal for durable responses; defining cellular composition can pinpoint immune components promoting anti-tumor activity and identify biomarkers associated with improved outcomes.

**Method:**

Regional lymph nodes (RLNs) were obtained at surgery from 29 patients with stage III melanoma. Eligible patients received adjuvant anti-PD-1 (αPD-1; pembrolizumab or nivolumab). Mass cytometry (CyTOF) was used to determine cellular composition of pre-treatment surgical specimens. NanoString bulk gene expression data from 125 patients receiving surgery without adjuvant therapy were used to evaluate trends observed in the CyTOF dataset.

**Results:**

Higher proportions of CD103^+^PD-1^+^CD8^+^ (T_RM_) T cells and plasmablast-like CD38^hi^CD19^dim^ cells were associated with improved prognosis in the CyTOF cohort. In the untreated cohort, a *“B cell excluded”* subgroup (< 2.5% tumor-infiltrating lymphocyte pathology score) had worse outcome, showing reduced B cell score and lower expression of activation genes including *CD38*, without change in CD8^+^ T cell score.

**Conclusion:**

Baseline infiltration of CD8^+^ T_RM_ and plasmablast-like CD38^hi^CD19^dim^ cells in RLN is strongly associated with prolonged distant metastasis-free survival in patients receiving αPD-1, supporting their potential as prognostic biomarkers in stage III melanoma.

## Introduction

Melanoma, although accounting for less than 5% of all skin cancers, represents the most lethal form of skin cancer (1). Early detection of local disease results in a high 5-year survival rate, ranging from 85-100% (1). Conversely, once melanoma progresses from the skin to the regional lymph nodes (stage III), the historical risk of relapse and subsequent development of distant metastases (stage IV) is high (2). This progression is associated with a drastically reduced 5-year survival, between 10-30%.

Immune checkpoint inhibitors (ICI) have revolutionized the treatment of metastatic melanoma, with long-term survival now achievable for more than half of patients (3). In stage III disease, adjuvant ICI substantially reduce the risk of relapse and distant metastasis, but cancer recurrence is still prevalent and treatment carries a risk of severe or permanent immune-related toxicity (4). Reliable tools to successfully predict benefit of treatment or which patients will develop distant metastases are therefore urgently needed. Several studies have highlighted the microenvironment as key to achieving an effective anti-tumoral response (5, 6). In particular, activated T cell populations have been emphasized as important drivers of response to ICI, while distinct cell subtypes/activation states have been associated with response (7–9). Nevertheless, the mechanisms associated with immune escape, treatment resistance and development of distant metastasis are not fully understood. Although several studies have attempted to identify biomarkers associated with poor prognosis (10–12), few studies have been performed, and no biomarkers have been established for stage III melanoma. By improving our understanding of tumor and microenvironmental changes in the context of ICI treatment and disease progression in stage III melanoma, novel prognostic and predictive biomarkers for response to ICI can be identified.

In the present study, biopsies from stage III melanoma were collected from regional lymph nodes to explore their immune- and tumor cell composition and further evaluate how their functional properties differ between patients developing distant metastasis following anti-PD-1 treatment (αPD-1) and those not presenting with metastatic disease.

## Materials and methods

### Patient cohort

Patients treated for stage III melanoma localized to the regional lymph node (RLN) at Oslo University Hospital (OUS) between 2020–2022 were included in the study cohort. Only patients with lymph node metastasis >1 cm were included in the study. Eligible patients received standard treatment consisting of surgery followed by αPD-1 therapy, administered as either pembrolizumab or nivolumab, for up to one year. For a subset of patients, adjuvant therapy was omitted because of old age, immunosuppressed condition, or poor compliance. Biopsies were collected from treatment-naïve patients at the time of the surgery.

### Tissue processing

Fresh tissue samples were first disintegrated into small pieces with a scalpel and then dissociated into single cell solution by enzymatic digestion using DMEM-F12 (Gibco, Life Technologies, Carlsbad, CA, USA) supplemented with 10% heat-inactivated fetal bovine serum (FBS), 50µg/ml DNase and 1 mg/ml Collagenase IV (all from Sigma-Aldrich, St.Louis, MO, USA) at 37 °C for 1 hour. The suspension was then passed through a 100 µm strainer and diluted in washing medium (DMEM-F12 supplemented with 2% FBS), followed by centrifugation at 400 g for 10 minutes. The samples were cryopreserved in aliquots of 2 × 10^6^ cells resuspended in DMEM-F12 supplemented with 70% FBS and 10% DMSO.

### CyTOF staining protocol

Cryopreserved samples were rapidly thawed in a 37 °C water bath, then mixed with pre-warmed thawing medium (RPMI 1640 (Gibco) supplemented with 10% FBS). The cells were centrifuged at 500 g for 5 min and resuspended in thawing media supplemented with 100µg/ml DNase, before being placed in an incubator at 37 °C for 30min. To identify live cells, cells were stained with Cisplatin in PBS (Gibco) for 5 min at room temperature, followed by incubation in FcR blocking solution (Human TruStain FcX™, BioLegend) to prevent non-specific binding. Extracellular antibodies (**Supplementary Table 1**) were mixed in the staining buffer containing 0.5 M EDTA (Invitrogen, Life Technologies) in Ca^2+^/Mg^2+^-free PBS supplemented with 1% sterile-filtered bovine serum albumin. Standard staining conditions were 100 µl antibody mix and 100 µl cell suspension containing ~1 × 10^6^ cells/ml. After incubation with antibodies for 30 minutes at 4 °C, cells were washed in 1 mL staining buffer. Cells were then fixed and permeabilized with eBioscience™ Foxp3/Transcription Factor staining buffer set (Invitrogen) before staining with antibodies against intracellular targets (**Supplementary Table 1**) following the manufacturer’s protocol. Finally, cells were incubated with the nucleic acid intercalator iridium (Standard BioTools) for 20 min, washed in Cell Acquisition Solution (CAS; Standard BioTools, South San Francisco, CA, USA) and filtered. Single cell suspensions stained with metal-conjugated antibodies were analyzed by Cytometry by Time-Of-Flight (CyTOF^®^) on the Helios™ system at the Flow Cytometry Core Facility, Montebello node at OUS. EQ™ Four Element Calibration Beads (Fluidigm) were added at 1:10 dilution immediately before sample injection into the CyTOF system. Batch effects were monitored by including three control samples in all separate CyTOF runs; PBMCs isolated from healthy individuals, and melanoma cell lines A-375 and MelMet5 melanoma cell lines.

### CyTOF data analysis

After acquisition, events were normalized, and calibration beads were removed using CyTOF software v7.0 (Standard BioTools). PeacoQC automated quality control to remove abnormal events was performed in CellMass Cytobank software (v6.0). Manual gating of live singlets on PeacoQC-controlled samples was then done based on four Gaussian parameters (center, offset, width and residual) and cisplatin (Live/Dead) and iridium (nuclear) staining. The quality controlled single live cell data were analyzed by FlowJo (v10.10.0, Ashland, OR, USA) and CytoBank softwares. Following data normalization and clean-up, the data from all samples were concatenated into a single file for UMAP dimension reduction analysis, before patient samples were re-identified in the concatenated file. Main cell populations were identified by lineage marker expression within the main UMAP clusters (**Supplementary Figure 1**), whereas sub-populations were gated manually (**Supplementary Figure 2**).

### CyTOF panel

To identify central cell populations within the RLN tumor-immune microenvironment, a 35-parameter antibody panel for characterization was established in-house. Metal-conjugated antibodies were used as described in (**Supplementary Table 1**), except for antibodies targeting Melan-A and AXL, where carrier-free antibodies were conjugated to metals using the Maxpar^®^ X8 Antibody Labeling Kit (Standard BioTools), according to the manufacturer’s protocol.

### FlowSOM analysis

Flow Self-Organizing Map (FlowSOM) (13) was used for unsupervised analysis of the CyTOF data. Prior to applying FlowSOM, the melanoma subpopulation was extracted from the concatenated dataset. FlowSOM was applied using default settings and 13 distinct cellular markers expressed in melanoma cells: CD47, AXL, CD80, ARG1, CD44, CD73, Melan-A, CD39, PD-L1, HLA-ABC, CD163, β-catenin and HLA-DR.

### Transcription analysis

Formalin-fixed paraffin-embedded (FFPE) blocks from regional lymph node metastases from patients with stage III metastatic melanoma who did not receive adjuvant treatment between 2010–2017 were retrieved from the archives at the Department of Pathology, OUS, and subjected to NanoString analyses. H&E diagnostic slides were used to select areas containing tumor and immune cells for each patient before RNA was extracted from the corresponding slides from a total of 125 samples. RNA was generated using the High Pure FFPET RNA isolation kit (Roche, Basel, Switzerland), before the nCounter PanCancer IO 360™ (IO360) panel was applied to all samples. Samples were run on the NanoString nCounter^®^ platform at the Experimental Diagnostics, Department of Pathology, OUS.

Quality checks were performed using the nSolver Analysis Software (v4.0.70), before counts were processed in R using the approach established by Bhattacharaya et al. (14).

### Statistical analysis and figure generation

Statistical analysis and figure generation were performed in the R (v4.3.1) programming language with RStudio (2023.09.0 + 463). Statistical significance was determined using the Wilcoxon non-parametric test in the R package *rstatix* (v0.7.2). p ≤ 0.05 was considered significant, and labeled with * p ≤ 0.05, ** p ≤ 0.01, *** p ≤ 0.001, **** p ≤ 0.0001.

### Survival analyses

Patients with distant metastasis at the time of surgery were excluded from survival analyses. Kaplan-Meier survival curves were generated using the R packages *survival* (v3.5-7) and *survminer* (v0.4.9), and differences between groups were assessed with the log-rank test. Univariable Cox proportional-hazards models were fitted for all clinicopathological variables in **Table 1**. Multivariable Cox models included CD103^+^PD-1^+^CD8^+^ T_RM_ and CD38^hi^CD19^dim^ cell proportion defined in this study, and Breslow depth, commonly used as a prognostic predictor in stage III melanoma. Ulceration was considered in a “full” model but excluded from the final model because of missing data for approximately one third of the patients. If events per variable were <5, Cox regression estimates were Firth-corrected using the *coxphf* package (v1.13.4).

**Table 1:** Clinicopathological parameters and associations with distant metastasis.

		All patients		Distant metastasis		No distant metastasis		*P*-value
Parameter		n	%	n	%	n	%	
Patients		29	100	17	59	12	41	
Age	Median (IQR)	71 (21)		71 (20)		69 (20.5)		0.60^a^
Sex	Female	10	34	4	40	6	60	0.24^b^
	Male	19	66	13	68	6	32	
Melanoma subtype	Superficial	8	50	5	63	3	38	0.57^b^
	Nodular	8	50	7	88	1	13	
Breslow	>2 mm	12	50	10	83	2	17	0.09^b^
	=<2 mm	12	50	5	42	7	58	
Ulceration	Yes	7	39	5	71	2	29	0.37^b^
	No	11	61	5	45	6	55	
Mutational status	BRAF	15	54	7	47	8	53	0.33^b^
	NRAS	5	18	4	80	1	20	
	None	8	29	6	75	2	25	
Mitotic index	Median (IQR)	7 (10)		10 (8.5)		4.5 (11.75)		0.79^a^
Checkpoint inhibitor treatment	Yes	22	76	13	59	9	41	1^b^
	No	7	24	4	57	3	43	
Survival	Alive	13	45	5	38	8	62	0.07^b^
	Dead	16	55	12	75	4	25	

^a^ Two sample Student's t-test.

^b^ Fisher's Exac.t

## Results

### Cellular composition of melanoma lymph node biopsies

**Figure 1:**
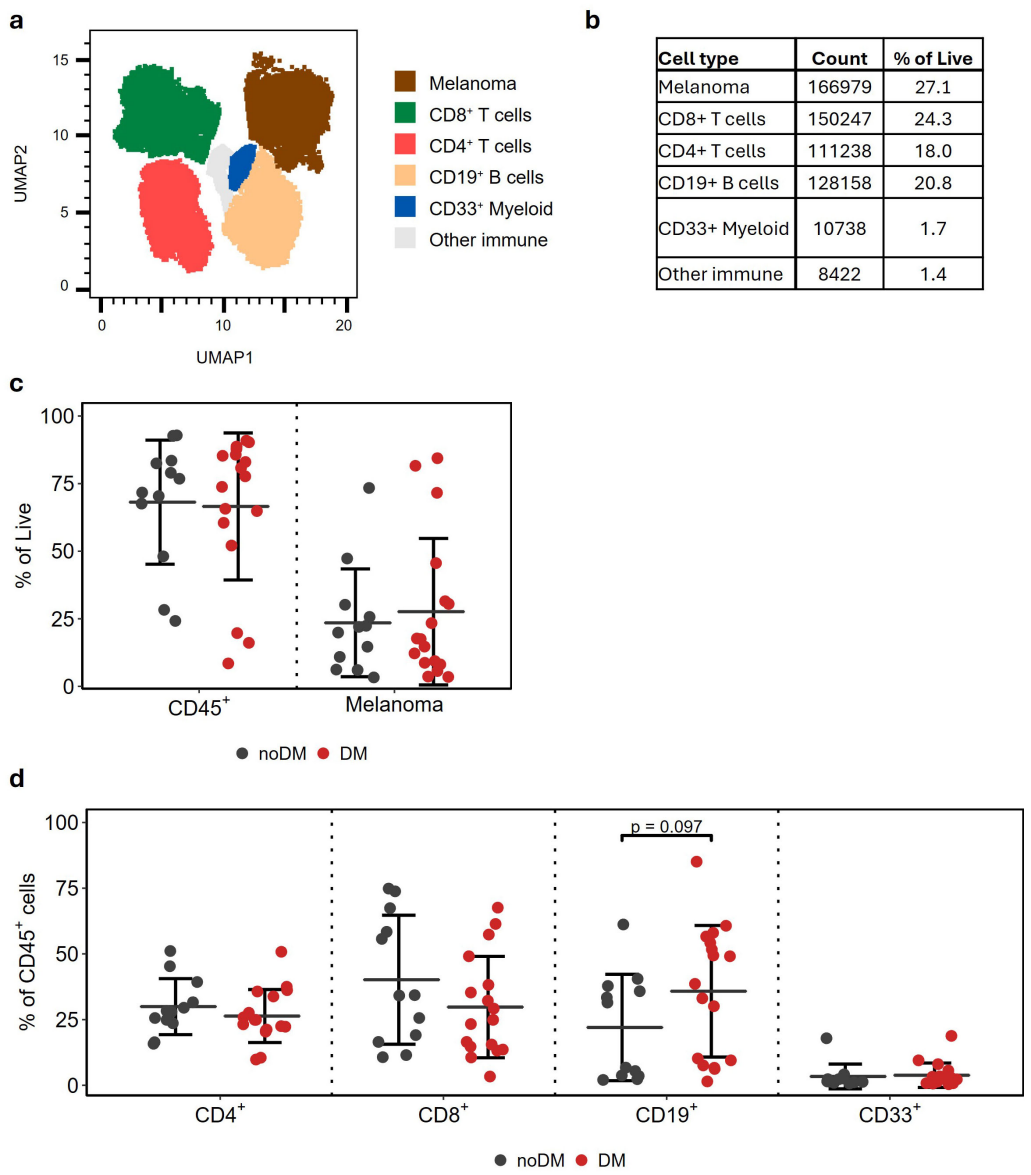
Cellular composition of melanoma lymph node biopsies. **(A)** UMAP of single cells from 29 lymph node biopsies from stage III melanoma. Main cell populations are highlighted. **(B)** Cell counts and corresponding mean frequencies for main cell populations in **(A, C)**. The relative proportion of melanoma and CD45^+^ immune cells as a percentage of all live cells in the corresponding biopsy. **(D)** The relative proportion of the major immune cell populations across biopsies. Mean ± SD as a fraction of CD45^+^ cells. noDM (n = 12); DM (n= 17).

To characterize melanoma metastasizing to the regional lymph node, we collected 29 biopsies from patients with operable stage III disease (**Table 1**). Initially, UMAP analyses were performed to obtain unbiased identification of tumor and immune cell composition (**Figure 1A**). This allowed for the identification of five major cell clusters, each contributing at least 1% of the total number of live cells (**Figure 1B**). Melanoma cells represented the largest population (27.1%), followed by CD8^+^ T cells (24.3%), CD19^+^ cells (20.8%), and CD4^+^ T cells (18%). We also identified minor populations of CD33^+^ myeloid cells (1.7%), as well as a smaller subset of other CD45^+^ cells (labeled “Other Immune”, ~1.4%).

Patients were grouped based on whether they developed distant metastasis (DM; n=17) or not (noDM; n=12) within the shortest follow-up available for patients without DM and still alive at the study conclusion (25.1 months; median follow-up of 33.5 months; **Supplementary Figure 3**). No differences were observed between the DM and noDM groups in the overall percentage of melanoma or CD45^+^ immune cells (**Figure 1C**). High heterogeneity was found in both groups, with tumors containing 8.5 to 92.9% CD45^+^ cells. Within CD45^+^ immune cells, a trend, though not significant, towards higher proportions of CD8^+^ T cells and lower fractions of CD19^+^ B cells was observed in the noDM group than the DM group (**Figure 1D**).

### No association between melanoma phenotype and DM

**Figure 2:**
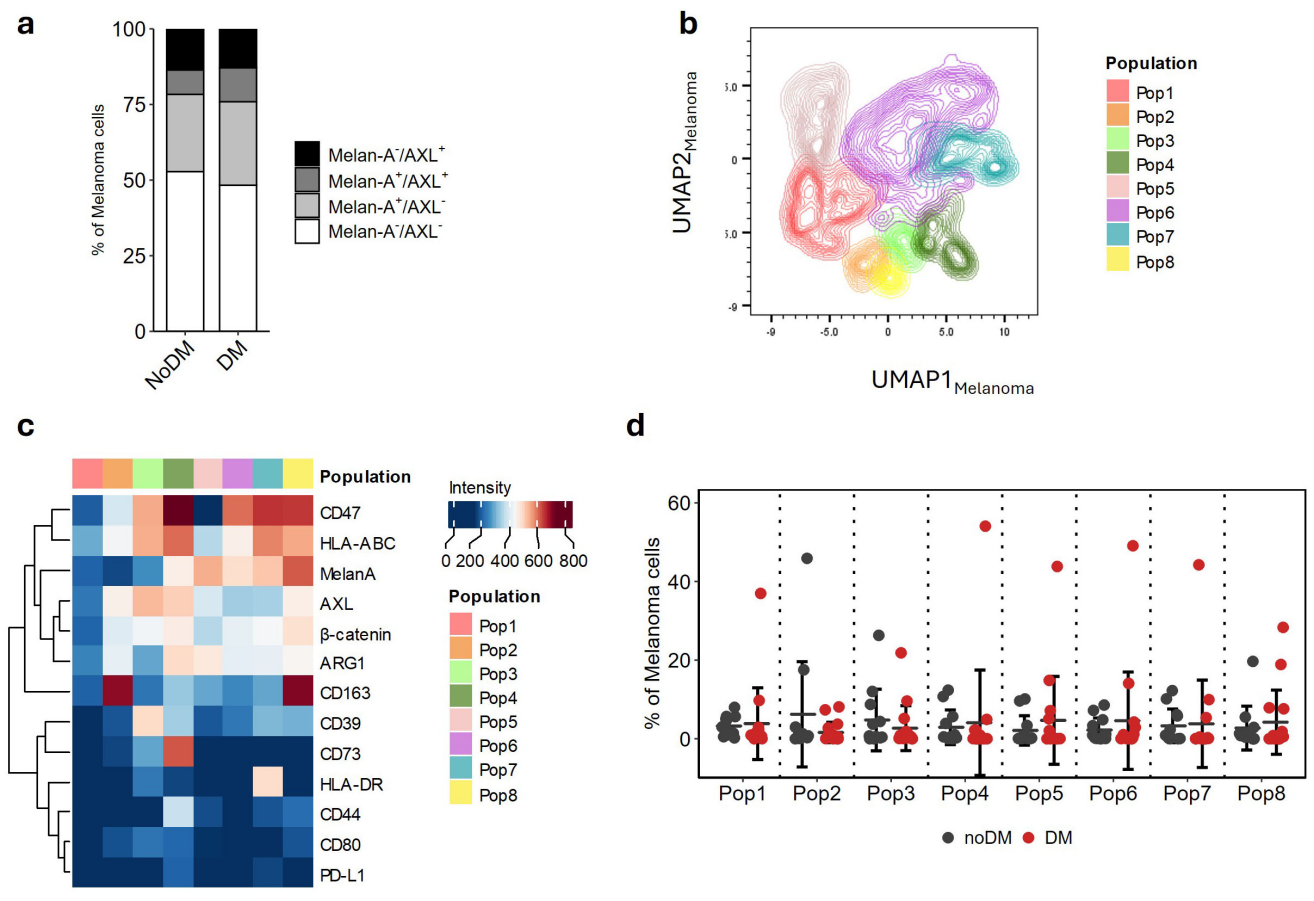
Melanoma phenotype is not associated with distant metastasis. **(A)** Mean proportion of Melan-A^-^/AXL^+^, Melan-A^+^/AXL^+^, Melan-A^+^/AXL^-^, Melan-A^-^/AXL^-^ across all biopsies. **(B)** UMAP of melanoma subcluster highlighting Population 1–8 identified by FlowSOM. **(C)** Populations in b clustered by their expression of cell markers used for FlowSOM analyses. **(D)** Populations/Clusters from b are shown as the relative proportion of melanoma cells represented by each sample.

No significant differences were observed in the proportion of cells expressing the established melanoma markers AXL and Melan-A (**Figure 2A**; **Supplementary Figure 4A**), or the correlation between their expression intensities (**Supplementary Figure 4B**) when the two groups were compared. To take advantage of high dimensional data, FlowSOM was used for unsupervised cluster identification across CD45^-^CD3^-^ melanoma cells. This allowed the identification of eight distinct cell populations within the melanoma cell cluster (**Figure 2B**). Each of these subpopulations were characterized by differences in the expression levels of distinct cellular markers (**Figure 2C**). Although large heterogeneity was observed across the samples, no significant differences were found when comparing the noDM and DM groups (**Figure 2D**).

### Trend towards higher PD-1 and activated T cell compartments in patients not developing distant metastases

**Figure 3:**
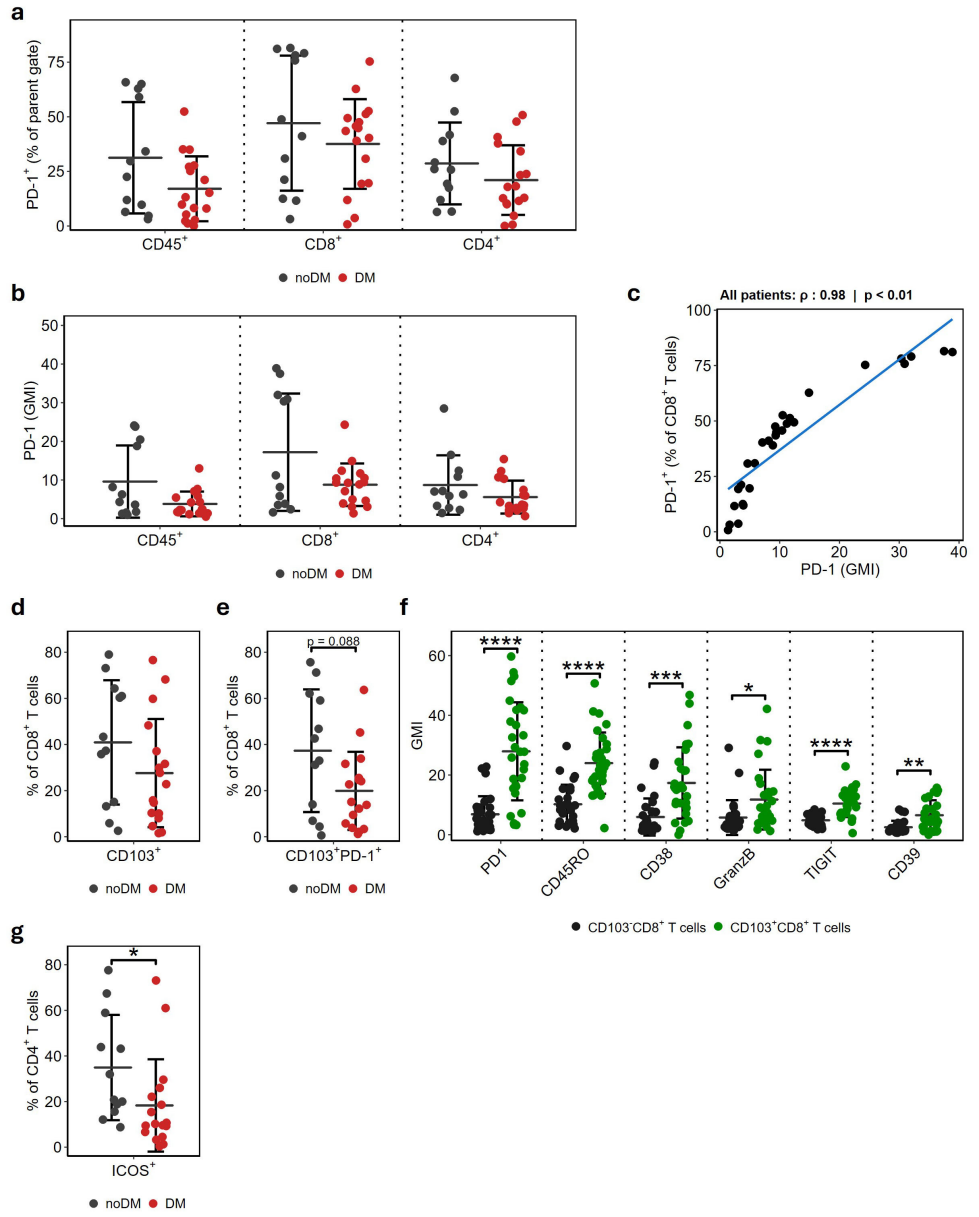
Biopsies from noDM patients are increased in activated phenotypes of CD8^+^ and CD4^+^ T cells. **(A)** Fraction of PD-1 positive cells within the CD8^+^ and CD4^+^ T cell populations, as well as across all CD45^+^ cells. PD-1 is represented as the proportion of positive cells in the respective population. **(B)** GMI for PD-1 in the same population as a. **(C)** Spearman correlation of PD-1^+^ proportion and intensity (GMI) in CD8^+^ T cells across all samples. **(D)** Proportion of CD103^+^ within the CD8^+^ T cell population. **(E)** GMI of surface markers in the CD103^+^ and CD103^-^ CD8^+^ T cell populations. **(F)** Proportion of CD103^+^PD-1^+^ cells within the CD8+ T cell population. **(G)** Proportion of ICOS^+^ cells within the CD4+ T cell population. Proportion of positive cells or GMI shown as mean ± SD. Asterisks denote statistical significance; * p ≤ 0.05, ** p ≤ 0.01, *** p ≤ 0.001, **** p ≤ 0.0001.

Since patients in this study cohort were subsequently treated with adjuvant αPD-1, we sought to evaluate whether the expression of PD-1 in tumor biopsies was associated with reduced risk of developing DM. PD-1 expression was mainly found on cells in the T cell compartment (CD8^+^ or CD4^+^; **Supplementary Figure 1**). We found a trend towards higher fraction of PD-1^+^ cells in the noDM group across all CD45^+^ cells, as well as CD8^+^ and CD4^+^ T cells (**Figure 3A**). As expression intensity is an important measure of downstream signaling, we assessed the geometric mean intensity (GMI) and observed a similar trend towards higher PD-1 expression in the noDM group for CD45^+^ cells, as well as the distinct T cell subsets (**Figure 3B**). Strong positive correlation between PD-1^+^ fraction and expression intensity was observed for CD45^+^ immune cells (**Supplementary Figure 4C**), CD8^+^ (**Figure 3C**) and CD4^+^ T cells (**Supplementary Figure 4D**).

We further assessed whether the slightly higher fraction of CD8^+^ T cells in the noDM group (**Figure 1D**) was associated with changes in immune cell activation status and checkpoint expression beyond PD-1. Tissue-resident memory (T_RM_) cells have been associated with improved survival in immunotherapy-naïve patients, and expand significantly during anti-PD-1 treatment (15). In line with this observation, a trend towards a higher fraction of T_RM_ defined by CD103^+^CD8^+^ expression was found in the noDM samples (**Figure 3D**; p = 0.166). Interestingly, the proportion of PD-1 expressing T_RM_ was approximately two times higher in the noDM group (**Figure 3E**; p = 0.088). Additionally, the T_RM_ population demonstrated a significant increase in the expression of CD45RO, CD38, Granzyme B, TIGIT and CD39 (**Figure 3F** and **Supplementary Figure 4F**), and a minor increase in HLA-DR, TIM-3 and LAG-3 (**Supplementary Figures 4E, F**), compared to CD103^-^CD8^+^ T cells, supporting an activated immune phenotype. Similarly, a significantly higher fraction of the activating marker ICOS^+^ (p = 0.018) was observed on CD4^+^ T cells in the noDM group (**Figure 3G**). Altogether, these data suggest the enrichment of an anti-tumoral microenvironment with activated T_RM_ and CD4^+^ T cells in the RLN of patients with noDM compared to DM.

### CD38^hi^CD19^dim^ cells are associated with improved distant metastasis-free survival

**Figure 4:**
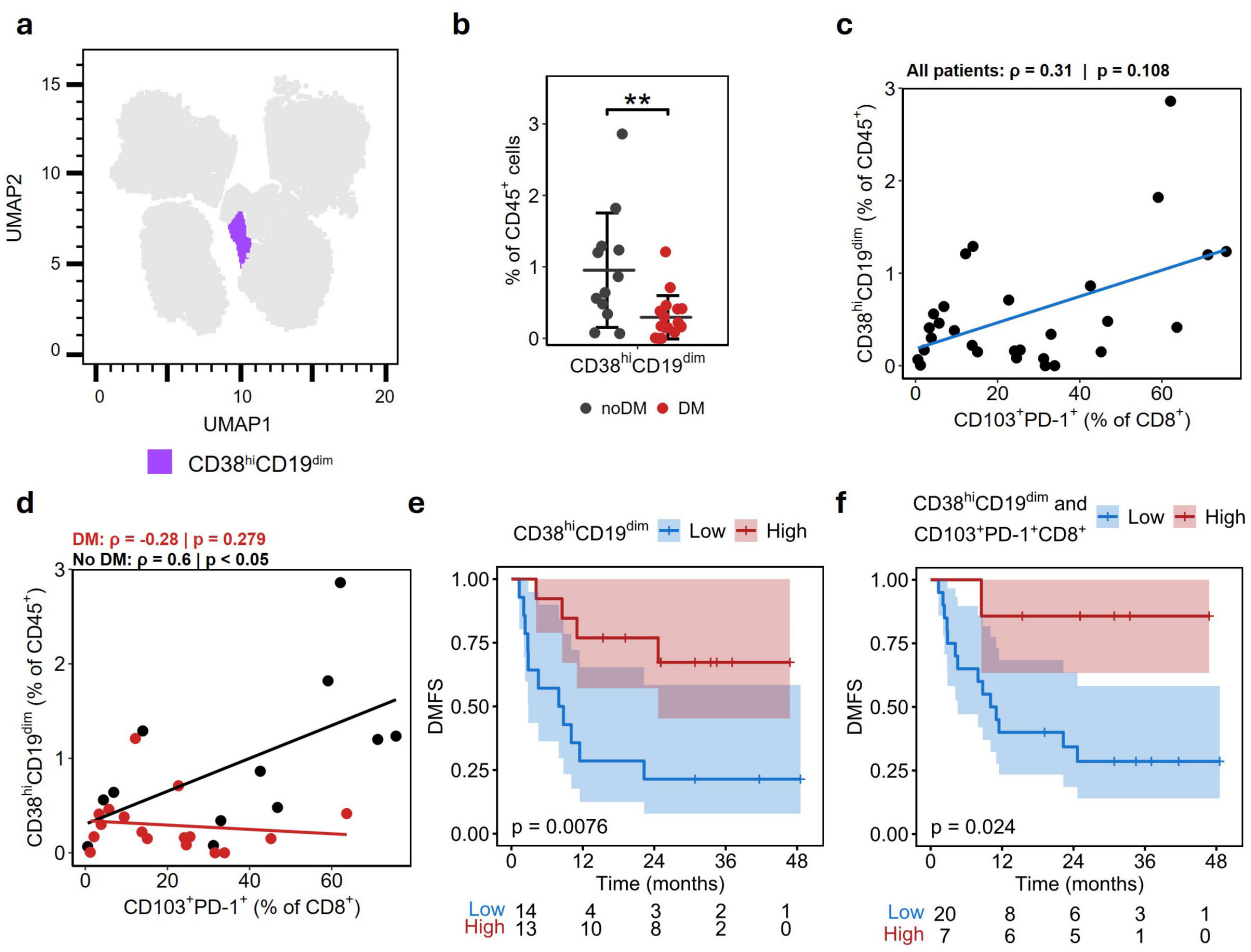
Reduced fraction of CD38^hi^CD19^dim^ associated with distant metastasis. **(A)** The cell population of CD19^dim^ cells with a corresponding increase in CD38 are highlighted in purple. **(B)** Proportion of CD38^hi^CD19^dim^ in DM and noDM groups relative to CD45^+^ cells. Mean ± SD. **(C, D)**. Spearman correlation of fraction of CD38^hi^CD19^dim^ and CD103^+^PD-1^+^CD8^+^ T cells, across all samples **(C)** and separated on DM-status **(D)**. **(E)** Kaplan-Meier plot showing distant metastasis-free survival (DMFS) in patients stratified on fraction of CD38^hi^CD19^dim^ higher (red) or lower (blue) than the median. **(F)** Kaplan-Meier plot stratified on fraction >median in both CD38^hi^CD19^dim^ and CD103^+^PD-1^+^CD8^+^ T cells, as *High*, remaining samples *Low*. P-values in e and f are calculated by log-rank test. Asterisks denote statistical significance; ** p ≤ 0.01.

No significant differences were found in CD19^+^ B cells between the DM and noDM groups, although there was a trend toward fewer CD19^+^ B cells in noDM samples (**Figure 1D**). Interestingly, by examining the “Other immune” cluster, a small fraction of CD38^+^ cells with intermediate levels of CD19-expression (labeled CD38^hi^CD19^dim^) were identified (**Figure 4A**; **Supplementary Figure 5A**). By comparing the DM-groups, a significantly higher fraction of CD38^hi^CD19^dim^ was found in patients without DM (**Figure 4B**). No association was observed between CD38^hi^CD19^dim^ and other clinicopathological variables (**Supplementary Table 2**), while only a moderate correlation was seen between CD38^hi^CD19^dim^ and CD103^+^PD-1^+^CD8^+^ T cells (**Figure 4C**). When separating samples according to DM-status, a strong positive correlation was found between the cell types only in noDM patients (**Figure 4D**). Similarly, an inverse correlation between CD38^hi^CD19^dim^ and CD19^+^ B cells was found only in tumors from the noDM group (**Supplementary Figures 4B, C**), while no strong correlation was found between CD38^hi^CD19^dim^ and ICOS^+^CD4^+^ T cells in either of the DM groups (**Supplementary Figures 5D, E**).

To explore whether these differences related to progression or potential long-term benefit from ICI, we stratified patients at the median into *High* and *Low* CD38^hi^CD19^dim^ groups (**Figure 4E**). The *High* group had significantly improved distant metastasis-free survival (DMFS) compared with the *Low* group (log-rank p-value = 0.0076), with the classifier showing an area under the ROC curve (AUC) of 0.81 with >90% specificity (**Supplementary Figure 5F**). Cox regression showed a significant association between higher CD38^hi^CD19^dim^ abundance and longer DMFS (Hazard ratio (HR) = 0.15, 95% CI 0.03 – 0.74; p < 0.02). Stratification by the CD103^+^PD-1^+^CD8^+^ T_RM_ subpopulation only defined a *High* expressing group doing marginally, though not significantly, better (**Supplementary Figure 5G**; p=0.09). In multivariable models adjusting for other covariates, only CD38^hi^CD19^dim^ remained statistically significant (p < 0.02; **Supplementary Table 3**). However, when making a surrogate for both markers (*High* group defined as >median expression of both cell populations), we identified a small group with excellent outcomes (only one distant metastasis during the 4-year follow-up; p = 0.026; **Figure 4F**).

### Loss of B cell score and CD38 expression associated with poor prognosis in stage III melanoma patients receiving no adjuvant therapy

**Figure 5:**
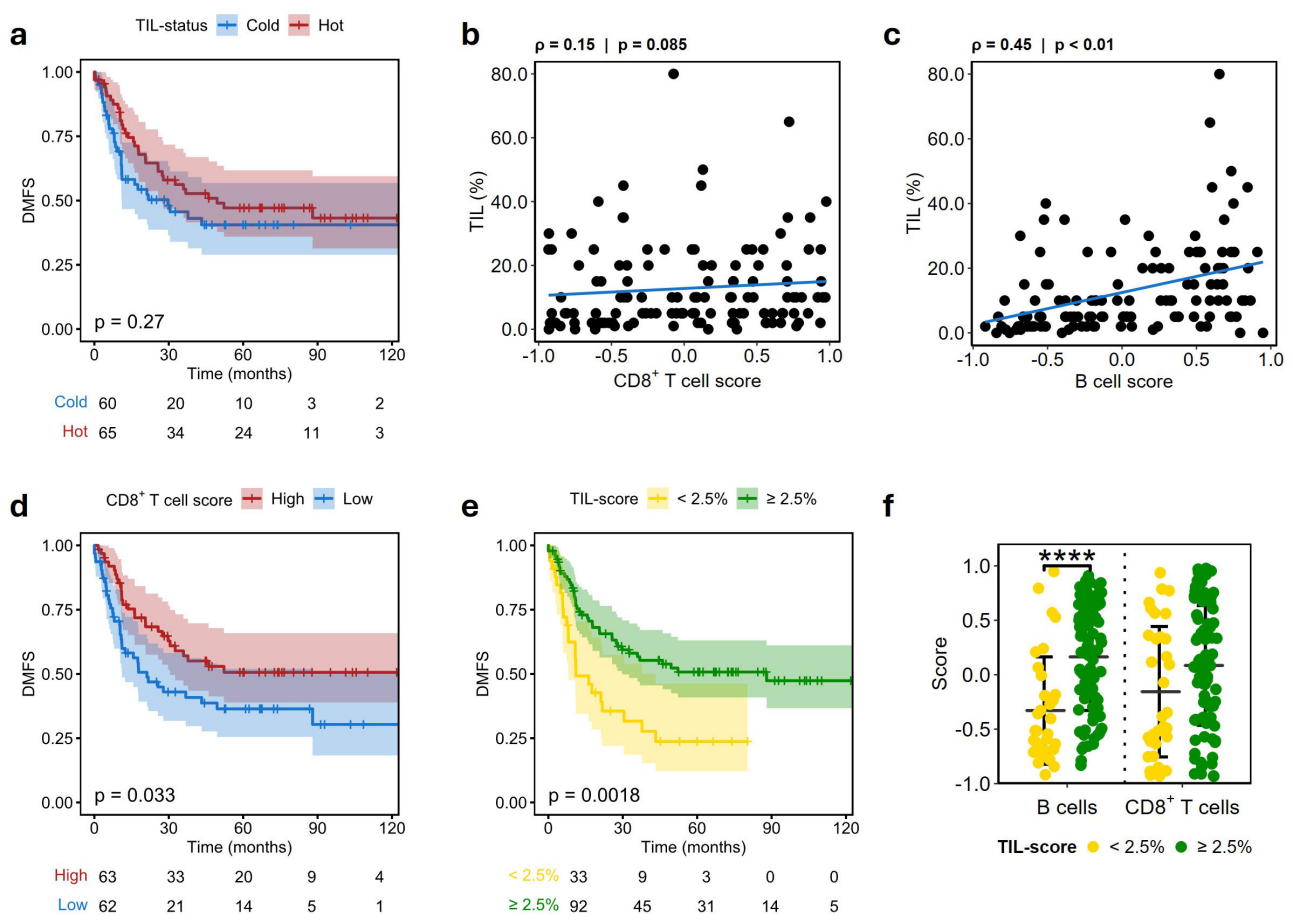
Low tumor-infiltrating lymphocytes are associated with reduced B cell score and poor outcome. **(A)** Kaplan-Meier plot showing distant metastasis-free survival (DMFS) in patients stratified on tumor-infiltrating lymphocytes (TIL), separated into Cold (<median infiltration) and Hot (>median infiltration). **(B, C)** Spearman correlation of fraction of TIL and CD8^+^ T cell **(B)** and B cell **(C)** gene scores from NanoString. **(D)** Kaplan-Meier plot showing DMFS in patients stratified on CD8^+^ T cell gene score from NanoString, separated by the median into *Low* and *High*. **(E)** Kaplan-Meier plot showing DMFS in patients stratified by < 2.5% pathologist TIL score. **(F)** B cell and CD8^+^ T cell gene score from NanoString in groups stratified by < 2.5% pathologist TIL score. Mean ± SD. P-values in a, d and e were calculated by log-rank test. Asterisks denote statistical significance; **** p ≤ 0.0001.

To further evaluate the prognostic impact of CD38^hi^CD19^dim^ and CD103^+^PD-1^+^CD8^+^ T cells, we utilized a NanoString dataset generated in-house from 125 regional lymph node biopsies collected from patients with stage III melanoma treated with surgery only. We first assessed how the tumor-infiltrating lymphocyte (TIL) score generated by pathological evaluation was associated with survival in this patient cohort. Interestingly, patients with Hot tumors (TIL score > median) did not show improved survival compared to patients with Cold tumors (**Figure 5A**). Using the cell subtype specific gene signatures provided with the IO360 panel, we proceeded to infer both CD8^+^ T cell and B cell scores, representing the two major lymphocyte populations found in the CyTOF cohort. No correlation was found between CD8^+^ T cell and TIL scores (**Figure 5B**), whereas a strong correlation was found between B cells and TIL scores (**Figure 5C**). By stratifying using the CD8^+^ T cell score inferred by NanoString, we found improved survival in patients with high CD8^+^ T cell score (p = 0.033; **Figure 5D**), but not when stratified by B cell score (**Supplementary Figure 6A**). By revisiting the cut-off for TIL infiltration, we identified a subset of patients with <2.5% TILs with reduced time to distant metastasis (p = 0.0018; **Figure 5E**). This “*B cell excluded*” group was associated with a highly significant drop in B cell score, but not in CD8^+^ T cell score (**Figure 5F**). As the B cell score in a bulk setting represents most B cell subsets, including plasmablast-like cells, we further analyzed the expression of selected markers linked to plasmablast-like and CD8^+^ T cell activation in this group. While the *B cell excluded* group had significantly reduced expression of markers associated with plasmablast-like cells; *CD38*, *CD27*, *CD19* and *CD79A/B*, no significant changes were observed for the genes *PDCD1* and *ITGAE*, encoding PD-1 and CD103, respectively (**Supplementary Figure 6B**). Notably, *CD38* and *CD19* expression were strongly correlated only in patients with TIL <2.5% (**Supplementary Figure 6C**) compared to *CD38* and *CD8A* which showed a similar correlation in the two groups (**Supplementary Figure 6D**).

## Discussion

In this study, we identified a subpopulation of CD38^hi^CD19^dim^ cells present at baseline that was associated with improved distant metastasis-free survival in melanoma patients receiving adjuvant treatment with αPD-1. Additionally, a higher fraction of CD38^hi^CD19^dim^ cells was strongly correlated with an increased proportion of CD103^+^PD-1^+^CD8^+^ cells in patients with noDM. We hypothesized that these subpopulations together generate an immune microenvironment beneficial for the effect of αPD-1 treatment. Evaluation of these subpopulations as predictive biomarkers of response to αPD-1 blockade could aid in the selection of patients who benefit from ICI.

Although melanoma cells were frequent in our cohort, we did not find specific phenotypes associated with disease progression and reduced response to ICI (**Figures 1A**, **2**). The relatively small cohort could partly explain this observation; however, melanoma is known to be heterogeneous, suggesting that the spatial context of the tumor clones could be necessary to fully understand the phenotypes associated with the treatment response or absence thereof (16). Similarly, only minor trends were observed in the major immune cell populations, as CD4^+^ and CD8^+^ T cells showed a slight decrease, and CD19^+^ B cells showed a slight increase in patients who developed DM (**Figure 1D**). This is in line with previous studies in metastatic melanoma, where the presence of CD8^+^ T cells has been reported in lesions responding to ICI (17).

In cutaneous melanoma, stem-like phenotypes are associated with metastatic spread and treatment resistance (18). This phenotype is often represented by high expression of the receptor tyrosine kinase AXL and low expression of melanocytic markers, such as Melan-A (19, 20). Consistent with observations in the present cohort (**Figure 2A**, **Supplementary Figures 4A, B**), we have previously shown that AXL is heterogeneously expressed in lymph node metastases from stage III melanoma (21) as well as in stage IV disease (22). Melanoma cells were mainly defined by CD45^-^CD3^-^, thus other cell types could potentially be assigned to this cluster. However, unsupervised analysis with FlowSOM did not identify subclusters associated with DM based on the markers available in our CyTOF panel. Altogether, this suggests that larger cohorts, in addition to spatial assessment, of RLN biopsies are necessary for further identification of melanoma phenotypes associated with the development of distant metastasis. Such studies would allow investigation of melanoma cell plasticity in a spatial context and may reveal a spectrum of activation states central to treatment escape and identify microenvironmental factors critical for response to ICI.

Although ICI have been highly successful in melanoma (3), there is still a lack of reliable biomarkers for predicting the benefit from therapy. Recent efforts utilizing -omics approaches have shown some promise for primary melanoma (11), but predictive biomarkers for stage III disease are still lacking. In a recent study, genes previously associated with ICI response (12) were used to generate an IFNγ-based signature with prognostic value in stage III melanoma but with limited predictive value of response to ICI (10). PD-L1 expression, evaluated by immunohistochemistry, is routinely used as a biomarker for PD-1/PD-L1 inhibitors in various cancers. However, in melanoma, this practice is less common due to considerable response rates, even in patients with PD-L1 negative tumors. Notably, the expression of PD-L1 has been shown to increase in melanoma LN metastasis compared to primary tumors (23). In our study, no significant association was found between PD-1 expression on CD45^+^ immune cells and development of distant metastasis in patients receiving αPD-1 (**Figures 3A–C**).

Several studies have highlighted the importance of different activation states of CD8^+^ T cells as predictors of response to immune therapy (6, 15, 24, 25). In the present study, we identified a higher proportion of CD103^+^CD8^+^ T cells, generally referred to as CD8^+^ T_RM_ cells, characterized by a high expression of the CD103 surface marker (26). CD8^+^ T_RM_ has been previously found at an increased frequency in melanoma lymph node metastases that do not develop into distant metastasis (6, 15). We found that CD8^+^ T_RM_ activation is characterized by increased expression of several activation markers (**Figure 3G**), including CD39, which has been associated with a favorable response to ICI when upregulated on CD8^+^ T_RM_ (25). Additionally, targeting the increased expression of surface markers, such as PD-1, CD38 and TIM-3, found on this population has been suggested to improve ICI sensitivity (15, 27–29). Altogether, this illustrates that a high pre-treatment proportion of activated T_RM_ with cytotoxic features generates a favorable microenvironment for effect of ICI therapy.

Interestingly, we found that CD8^+^ T cell gene score, but not pathologist-generated TIL score, was associated with good prognosis in our no-adjuvant cohort (**Figure 5**). The TIL score itself seemed to most accurately represent B cell infiltration rather than CD8^+^ T cells. Lower B cell infiltration was also observed in the CyTOF data (**Figure 1D**) of patients with noDM after ICI, but B cell score was not directly linked to prognosis in the NanoString cohort. Others have observed CD19^+^ B cells to be associated with good outcome in stage III melanoma, but it is worth noting that their study was performed in stage III melanoma collected from skin and RLN (30), representing a slightly different cohort than that used in this study. Reports indicate that impaired B cell activation serves as a step in the development of advanced melanoma (31), and the clonal B cell receptor (BCR) repertoire is a good indicator of response to ICI in metastatic melanoma (32). Altogether this suggests TIL score on its own to be of limited use when assessing the tumors immune-status in the context of likely ICI response, as both T cell and B cell activation states are important factors for accurately assessing immune activation.

We identified a small population of CD38^hi^CD19^dim^ cells that were strongly associated with good prognosis after ICI treatment. This subpopulation showed marker expression in line with that of plasmablast-like cells (33), with high expression of CD38, low expression of CD19, and absence of T cell lineage markers (**Supplementary Figure 5A**). Interestingly, the increased proportion of these plasmablast-like cells before ICI strongly correlated with longer time until distant metastasis and showed improved prognostic impact compared to the PD-1^+^ CD8^+^ T_RM_ fraction alone (**Figure 4E**, **Supplementary Figure 5G**, **Supplementary Table 3**). Patients with high fractions of both PD-1^+^ CD8^+^ T_RM_ and CD38^hi^CD19^dim^ showed even better outcome, with only 1/7 patients developing distant metastasis. In addition, the fraction of these subtypes showed strong correlation in samples from patients who did not develop DM (**Figure 4D**), but not in patients who developed DM, indicating the potential importance of both these cell types in defining a favorable microenvironment for response to ICI. In line with our observation, Griss et al. observed plasmablast-like (CD38^+^CD19^+^) cells to be central in shaping the microenvironment in cutaneous melanoma, and to be associated with increased PD-1^+^ T cell activation after anti-PD-1 blockade *in vitro* (33). Additionally, they found that higher frequency of these plasmablast-like cells predicted response to immune checkpoint blockade and survival in metastatic melanoma (stage IV), supporting the presence of an anti-tumoral immune microenvironment reinvigorated by ICI therapy. The tumor-induced plasmablast-like-enriched B cell population (TIPB) signature defined in the Griss study, consisting of *CD27*, *CD38*, and *PAX5*, needs to be refined in a bulk tumor setting. For instance, although CD38 is a prominent marker on plasmablast-like cells, it is also expressed in multiple cell types, which can influence its overall expression in bulk samples. Notably, a high proportion of dysfunctional PD-1^+^CD38^hi^ CD8^+^ T cells has been reported to be both a predictive and therapeutic biomarker for anti-PD-1 treatment (29). This underscores the importance of accounting for the phenotype and activation status of various immune cells when defining novel biomarkers and highlights the advantage of using single cells rather than bulk signatures as prognostic markers. Furthermore, Griss et al. reported a strong correlation between TIPB and multiple signatures of T cells and cytotoxic activity, suggesting that TIPB is significantly influenced by CD8^+^ T cell expression. Using NanoString gene expression data from a cohort of untreated patients, we assessed the expression of genes associated with plasmablast-like genes and B cell scores. While, in line with the CyTOF data, high CD8^+^ T cell scores were associated with improved survival in patients not receiving adjuvant therapy, B cell score alone were not associated with disease prognosis. Interestingly, a subset of *B cell excluded* tumors with reduced lymphocyte infiltration (TIL < 2.5%) was strongly correlated with worse outcome and a significant reduction in B cell scores. This was accompanied by reduced expression of both *CD38* and *CD27*, genes central to the plasmablast-like phenotype, but not reduction in CD8^+^ T cell score. We hypothesized that the absence of plasmablast-like cells is central to the poor prognosis of this group. Similarly, we found strong correlation between *CD19* and *CD38* only in this subset of patients, suggesting that the decrease in *CD38* was due to changes in B cell presence rather than CD8^+^ T cells, highlighting the potential for using plasmablast-like cells as stratification criteria in stage III melanoma. Our study demonstrates how single cell approaches can enhance the stratification of these patients relative to bulk approaches.

While this study identified several cell types associated with favorable prognosis in patients receiving adjuvant αPD-1 therapy, it is important to highlight that without any treatment response measure, we are unable to conclude whether these features can directly predict response to αPD-1 treatment. Notably, we observed similar associations between CD8^+^ T cell scores, genes associated with plasmablast-like cells and good prognosis among patients who did not receive adjuvant treatment in the NanoString cohort. This suggests that enrichment of these cells prior to therapy is indicative of a better outcome, regardless of adjuvant treatment. Additionally, the current standard-of-care for stage III melanoma includes neo-adjuvant αPD-1 therapy before lymph node resection. Collecting appropriate specimens in this context could help to evaluate whether T_RM_ and plasmablast-like cells serve as predictive markers for αPD-1 treatment according to the current standard-of-care, while also identifying patients with favorable prognoses, as the current study suggests.

## Conclusion

In this study, several immune cell types in the RLN were found to be associated with better prognosis in stage III melanoma treated with adjuvant αPD-1 after lymph node surgery. Notably, high fractions of CD38^hi^CD19^dim^ plasmablast-like cells and activated CD103^+^PD-1^+^CD8^+^ T_RM_ were strongly associated with distant metastasis-free survival. While the findings of this explorative study require validation in an independent patient cohort, it highlights the potential for using immune cell subsets as prognostic biomarkers and possibly for predicting response to αPD-1 in this patient group.

## Data Availability

The original contributions presented in the study are included in the article/**Supplementary Material**. Further inquiries can be directed to the corresponding author.
