# Supplementary material for: CD38hiCD19dim cells in lymph nodes predict favorable prognosis in patients with stage III melanoma receiving adjuvant PD-1-blockade
Source: Front Oncol. 2026 May 4;16:1815008. doi: 10.3389/fonc.2026.1815008 (PMC13180574; doi:10.3389/fonc.2026.1815008)
Supplement: Supplementary file 1 [file DataSheet1.pdf]

Supplementary Table S1

List of antibodies

Target	Mass	Metal	Clone	Manufacturer	Catalog #	Final concentration	Additional Info
**CD45**	89	Y	HI30	Standard BioTools	3089003C	1:400	
**CD19**	142	Nd	HIB19	Standard BioTools	3142001C	1:400	
**ICOS**	143	Nd	C398.4A	Standard BioTools	3143025C	1:400	
**HLA-ABC**	144	Nd	W6-32	Standard BioTools	3144017C	1:400	
**CD163**	145	Nd	GHI/61	Standard BioTools	3145010B	1:200	Intracellular
**CD8a**	146	Nd	RPA-T8	Standard BioTools	3146001C	1:400	
**β-catenin**	147	Sm	D10A8	Standard BioTools	3147005C	1:400	Intracellular
**CD16**	148	Nd	3G8	Standard BioTools	3148004B	1:200	
**CD25**	149	Sm	2A3	Standard BioTools	3149010C	1:400	
**LAG3**	150	Nd	11C3C65	Standard BioTools	3150030B	1:200	
**CD103**	151	Eu	Ber-ACT8	Standard BioTools	3151011B	1:200	
**TCRgd**	152	Sm	11F2	Standard BioTools	3152008B	1:200	
**TIM3**	153	Eu	F38-2E2	Standard BioTools	3153008B	1:200	
**TIGIT**	154	Sm	MBSA43	Standard BioTools	3154016B	1:200	
**PD-1**	155	Gd	EH12.2H7	Standard BioTools	3155009B	1:200	
**CD14**	156	Gd	HCD14	Standard BioTools	3156019B	1:200	
**Melan-A**	158	Gd	#872719	R&D systems	MAB8008	1:200	Intracellular/Conjugate
**CD39**	160	Gd	A1	Standard BioTools	3160004C	1:400	
**AXL**	161	Dy	Polyclonal	R&D systems	AF154	1:400	Intracellular/Conjugate
**CD80**	162	Dy	2D10.4	Standard BioTools	3162010B	1:200	
**CD33**	163	Dy	WM53	Standard BioTools	3163023C	1:400	
**Arginase-1**	164	Dy	14D2C43	Standard BioTools	3164030B	1:200	Intracellular
**CD45RO**	165	Ho	UCHL1	Standard BioTools	3165011C	1:400	
**CD44**	166	Er	BJ18	Standard BioTools	3166001C	1:200	
**CD11b**	167	Er	ICRF44	Standard BioTools	3167011C	1:400	
**CD73**	168	Er	AD2	Standard BioTools	3168015B	1:200	
**CD45RA**	169	Tm	HI100	Standard BioTools	3169008B	1:400	
**CD3**	170	Er	UCHT1	Standard BioTools	3170001C	1:400	
**Granzyme B**	171	Yb	GB11	Standard BioTools	3171002C	1:200	Intracellular
**CD38**	172	Yb	HIT2	Standard BioTools	3172007C	1:200	
**HLA-DR**	173	Yb	L243	Standard BioTools	3173005C	1:400	
**CD4**	174	Yb	SK3	Standard BioTools	3174004C	1:400	
**PD-L1**	175	Lu	29E.2A3	Standard BioTools	3175017B	1:200	
**CD56**	176	Yb	N901	Standard BioTools	3176009C	1:200	
**CD47**	209	Bi	CC2C6	Standard BioTools	3209004C	1:200	

Supplementary Table S2

Clinicopathological parameters and associations with CD38^{hi}CD19^{dim} cells

		CD38^{hi}CD19^{dim} >= median		*P*-value
		n	%	
**Patients**		14	48	
**Age**	Median (IQR)	69 (19)		0.91[a]
**Sex**	Female	4	40	0.7[b]
	Male	10	53	
**Melanoma subtype**	Superficial	3	38	1[b]
	Nodular	3	38	
**Breslow**	>2 mm	4	33	0.41[b]
	=<2 mm	7	58	
**Ulceration**	Yes	3	43	1[b]
	No	5	46	
**Mutational status**	BRAF	7	47	1[b]
	NRAS	2	40	
	None	4	50	
**Mitotic index**	Median (IQR)	7 (10)		1[a]
**Checkpoint inhibitor treatment**	Yes	10	46	0.68[b]
	No	4	57	
**Survival**	Alive	7	44	0.87[c]
	Dead	7	54	
**Distant Metastasis**	DM	5	29	**0.03**[b]
	noDM	9	75	

[a]Two sample Student's t-test

[b]Fisher's Exact

[c]Chi-squared

Supplementary Table S3

Covariate analyses

	Crude				Adjusted (full)				Adjusted (final)[c]			
Predictor	Patients (n)	Events (n)	HR	P-value	Patients (n)	Events (n)	HR[b]	P-value	Patients (n)	Events (n)	HR[b]	P-value
CD38^{hi}CD19^{dim}	27	15	0.15[a] (0.03-0.73)	***0.02***	18	10	0.15 (0.02-1.31)	***0.02***	22	13	0.11 (0.01-0.94)	***0.01***
PD1^{+}CD8^{+} T$_{RM}$	27	15	0.98[a] (0.95-1.00)	0.07	18	10	1.01 (0.97-1.05)	0.52	22	13	1.00 (0.97-1.03)	0.98
Breslow (mm)	22	13	1.04 (0.96-1.13)	0.31	18	10	1.00 (0.91-1.10)	0.99	22	13	1.00 (0.91-1.09)	0.91
Ulceration (Yes)	18	10	1.96 (0.56-6.87)	0.29	18	10	1.72 (0.42-7.01)	0.42				
Mitotic index	13	7	1.02 (0.92-1.12)	0.75								
Age	27	15	0.99 (0.96-1.02)	0.60								
Sex (Female)	27	15	0.51 (0.16-1.61)	0.25								
Mutational status (BRAF vs None)	26	15	0.48 (0.15-1.53)	0.22								
Mutational status (NRAS vs None)	26	15	0.99 (0.24-4.17)	0.99								
Checkpoint inhibitor treatment (Yes)	27	15	0.92 (0.29-2.93)	0.89								
Melanoma subtype (Superficial)	15	11	0.59 (0.18-1.95)	0.38								

[a] HR per 1 percentage-point increase in cell proportion

[b] Firth-penalized Cox estimates

[c] Ulceration was excluded in the final model due to many missing values
